# Predicting Anticipated Telehealth Use: Development of the CONTEST Score and Machine Learning Models Using a National U.S. Survey

**DOI:** 10.3390/healthcare14040500

**Published:** 2026-02-14

**Authors:** Richard C. Wang, Usha Sambamoorthi

**Affiliations:** 1 St. Mark’s School of Texas, 10600 Preston Rd., Dallas, TX 75230, USA; 2 Department of Pharmacotherapy, Texas Center for Health Disparities, College of Pharmacy, University of North Texas Health Science Center, 3500 Camp Bowie Blvd., Fort Worth, TX 76107, USA; usha.sambamoorthi@unthsc.edu

**Keywords:** telehealth, machine learning, fairness, HINTS

## Abstract

**Objectives:** Anticipated telehealth use is an important determinant of whether telehealth can function as a durable component of hybrid care models. However, there are limited practical tools to identify patients at risk of discontinuing telehealth. We aim to (1) identify factors associated with anticipated telehealth use; (2) develop a risk stratification tool (CONTEST); (3) compare its performance with machine learning (ML) models; and (4) evaluate model fairness across sex and race/ethnicity. **Methods:** We conducted a retrospective cross-sectional analysis of the 2024 Health Information National Trends Survey 7 (HINTS 7), including U.S. adults with ≥1 telehealth visit in the prior 12 months. The primary outcome was anticipated telehealth use. Survey-weighted multivariable logistic regression informed a Framingham-style point score (CONTEST). ML models (XGBoost, random forest, logistic regression) were trained and evaluated using the area under the receiver operating characteristic curve (AUROC), precision, and recall. Global interpretation used SHAP values. Fairness was assessed using group metrics (Disparate Impact, Equal Opportunity) and individual counterfactual-flip rates (CFR). **Results:** Approximately one-third of adults reported at least one telehealth visit in the prior year. Among these users, nearly one in ten expressed an unwillingness to continue using telehealth in the future. Four telehealth experience factors were independently associated with unwillingness to continue: lower perceived convenience, technical problems, lower perceived quality compared to in-person care, and unwillingness to recommend telehealth. CONTEST demonstrated strong discrimination for identifying individuals with lower anticipated telehealth use (AUROC 0.876; 95% CI, 0.843–0.908). XGBoost performed best among the ML models (AUROC 0.902 with all features). With the same four top features, an ML-informed point score achieved an AUROC of 0.872 (95% CI, 0.839–0.904), and a four-feature XGBoost model yielded an AUROC of 0.893 (95% CI, 0.821–0.948, *p* > 0.05). Group fairness metrics revealed disparities across sex and race/ethnicity, whereas individual counterfactual analyses indicated low flip rates (sex CFR: 0.024; race/ethnicity CFR: 0.013). **Conclusions:** A parsimonious, interpretable score (CONTEST) and feature-matched ML models provide comparable discrimination for stratifying risk of lower anticipated telehealth use. Sustained engagement hinges on convenience, technical reliability, perceived quality, and patient advocacy. Implementation should pair prediction with operational support and routine fairness monitoring to mitigate subgroup disparities.

## 1. Introduction

Telehealth, which encompasses synchronous video and telephone communication, has emerged as an essential modality for delivering clinical care across various settings [1,2]. In addition to substituting for traditional in-person encounters, telehealth serves as an adjunct pathway that can expand access by overcoming geographic, transportation, mobility, and scheduling barriers [3,4]. The COVID-19 pandemic catalyzed a rapid, system-wide shift toward virtual care, with many organizations developing the infrastructure, clinical protocols, and reimbursement workflows necessary to sustain telehealth beyond the acute public health emergency. Since then, health systems and payers have continued to integrate telehealth into routine care pathways, either as a first-line option for appropriate conditions or as a complementary touchpoint within hybrid care models [3,5]. Recent national survey data from 2022 suggest that approximately 39% of U.S. adults had a telehealth visit in the preceding year [6]. In the post-COVID era, utilization remains substantial, making this modality a stable component of healthcare delivery in the United States [7,8].

From clinical and operational standpoints, telehealth offers several advantages. Adding a flexible channel for care can shorten the time to evaluation, facilitate follow-up, and support chronic disease management between in-person visits [3,4,9]. At the system level, virtual care may increase clinical throughput, optimize clinician scheduling, and extend scarce specialty resources across broader catchment areas [10,11]. For patients, telehealth can lower both direct and indirect costs, including travel, childcare, time away from work, and lost wages, thereby reducing the overall burden of seeking care [12,13]. More broadly, when appropriately targeted, telehealth encounters can help avert some urgent care and emergency department utilization, conserving high-acuity resources for those who are most in need [14].

Despite these advantages, concerns about the quality and acceptability of telehealth persist. Patients and clinicians have cited limitations, including the absence of a physical examination, variable audio–video quality, and connectivity requirements that presuppose reliable Internet access and a compatible device [15,16]. Communication dynamics may differ in virtual settings, potentially affecting rapport, shared decision-making, and diagnostic confidence [17]. Some patients prefer traditional in-person visits for reassurance, hands-on assessment, or cultural and personal reasons; others encounter digital literacy barriers or accessibility challenges (e.g., language barriers) [18,19,20]. On the provider and system sides, integration with electronic health records, workflow alignment, licensure and reimbursement rules, and privacy/security considerations can all shape utilization patterns [21,22]. While individual studies have documented elements of patient dissatisfaction or a preference for in-person care, the determinants of sustained telehealth use, especially at the individual patient level, remain incompletely characterized [15,23,24].

A critical gap is the absence of validated tools to identify which patients are likely to continue using telehealth after the initial use (i.e., anticipated telehealth use). Existing telemedicine evaluation instruments, such as the Telehealth Usability Questionnaire (TUQ), Telemedicine Satisfaction Questionnaire (TSQ), and Service User Technology Acceptability Questionnaire (SUTAQ), primarily assess usability, satisfaction, or acceptance following a telehealth encounter, and are intended for retrospective evaluation rather than prospective risk stratification [25]. More recently, the Video Visit User Experience (VVUE) score demonstrated an association between patient experience and subsequent video visit use [26]. However, it remains an experience-based measure rather than a screening tool explicitly designed to identify individuals at risk of disengagement. As a result, these approaches are limited in their ability to support proactive, operational decision-making [8,25,26,27].

A predictive framework for anticipated telehealth use would have practical value. It could guide clinicians in recommending the most acceptable and effective modality and help health systems design hybrid pathways that reduce avoidable in-person and emergency department visits, thereby mitigating crowding and improving resource allocation. Therefore, tools that anticipate telehealth use are most relevant to three audiences: (1) clinicians and care teams deciding whether telehealth is appropriate for follow-up or chronic disease management; (2) telehealth program leaders and health systems designing hybrid pathways and allocating operational support (e.g., onboarding and technical assistance); and (3) researchers developing interpretable and equitable prediction models for deployment [21,22]. Although prior work has examined telehealth adoption, satisfaction, and utilization patterns, fewer studies have focused on an individual’s likelihood of continuing telehealth use after trying it, and even fewer have provided deployable risk-stratification tools that balance interpretability, predictive performance, and equity considerations [21,28]. In contrast, a parsimonious screening score, such as CONTEST, is designed to integrate multiple experiential dimensions into a single, interpretable risk-stratification tool that can be applied at the point of care or at program enrollment to guide targeted support, rather than to summarize satisfaction after the fact.

To address these gaps, this study used nationally representative U.S. survey data to develop and evaluate complementary predictive approaches. Specifically, we aim to: (1) identify sociodemographic and telehealth-experience factors associated with anticipated telehealth use; (2) develop a parsimonious, interpretable point-based risk stratification tool to identify individuals at risk of discontinuation; (3) compare its predictive performance with commonly used machine learning (ML) models; and (4) evaluate model fairness across sex and race/ethnicity using group- and individual-level metrics. By advancing both an interpretable score and a rigorously evaluated ML model, including global interpretation and fairness evaluation, this study provides a practical, evidence-informed foundation for optimizing telehealth within hybrid care models, improving patient experience, and enhancing access while safeguarding equity and quality.

## 2. Methods

### 2.1. Study Design and Data Retrieval

This retrospective cross-sectional study analyzed data from the Health Information National Trends Survey 7 (HINTS 7). HINTS, administered by the National Cancer Institute, is a nationally representative survey designed to track changes in health communication. The dataset is publicly available and fully deidentified. Data collection for HINTS 7 took place from 25 March 2024 to 16 September 2024, resulting in a final sample of 7278 respondents. Because the dataset is public and de-identified, this analysis was deemed non-human subject research and exempt from the University of North Texas Health Regional Institutional Review Board (IRB, No 2368162-1). Therefore, informed consent was not required.

### 2.2. Inclusion and Exclusion Criteria

We included U.S. adults who reported at least one telehealth visit in the preceding 12 months. We excluded respondents who (1) reported no telehealth use during that period; (2) did not answer the item assessing anticipated telehealth use; (3) were missing key telehealth variables (e.g., technology challenges, perceived quality of care, reasons for telehealth use); or (4) lacked essential sociodemographic information (e.g., age, sex, marital status, race/ethnicity).

### 2.3. Primary Outcome

Our primary outcome was the respondents’ anticipated telehealth use, measured with the survey item: “How willing are you to do a telehealth visit in the future if one is offered to you?” Response options were “very willing”, “somewhat willing”, “somewhat unwilling”, and “very unwilling”. Anticipated telehealth use was selected as the primary outcome because it reflects respondents’ forward-looking expectations following a prior telehealth experience. While anticipated use does not directly measure realized utilization, it is commonly used in survey-based health services research when longitudinal follow-up is unavailable. For analysis, we dichotomized this item to indicate willingness to continue telehealth (“yes”: very willing or somewhat willing) versus unwillingness (“no”: somewhat unwilling or very unwilling). We dichotomized the outcome to distinguish individuals who are willing from those who are unwilling to continue telehealth use to support the development of an interpretable screening and risk-stratification tool. While this approach may result in information loss, it facilitates clinical translation and comparison between point-based and ML models.

### 2.4. Key Variables

Key covariates encompassed the respondents’ sociodemographic characteristics and telehealth experiences. Sociodemographic variables included age (18–34, 35–49, 50–64, 65–74, ≥75 years), sex (male, female), race/ethnicity (non-Hispanic White, non-Hispanic Black, Hispanic, other), marital status (single, married, other), education (less than high school, high school graduate, some college, college graduate or higher), household income (<$50,000; $50,000–$99,999; ≥$100,000), and health insurance coverage (yes/no).

Telehealth experience was captured with 10 dichotomous (yes/no) items: (1) provider recommendation or requirement to use telehealth (refers to as recommend); (2) desire for advice about whether in-person care was needed (refers to as advice); (3) avoidance of possible infection in clinical settings (refers to as avoidance); (4) greater convenience than visiting a clinician in person (refers to as convenience); (5) lack of local availability of a health professional (refers to as availability); (6) ability to include family or other caregivers in the appointment (refers to as involvement); (7) technical problems during the telehealth visit (refers to as technique); (8) perception that telehealth care quality was comparable to an in-person visit (refers to as quality); (9) telehealth made it easier to obtain care when and where needed (refers to as access); and (10) willingness to recommend telehealth to others (refers to as self-recommend). We also included the primary reason for the most recent telehealth visit (annual checkup, minor illness/acute care, chronic condition management, mental health, other).

### 2.5. Statistical Analysis and Derivation of the Scoring System

Survey respondents were classified into two groups based on their stated willingness to continue using telehealth. For all variables, we estimated weighted percentages to account for the complex survey design. Sociodemographic characteristics and telehealth experiences were compared between groups using Rao–Scott χ^2^ tests. We then fit multivariable, survey-weighted logistic regression models to identify factors associated with willingness to continue telehealth, adjusting for sociodemographic and telehealth experience variables. Results are reported as adjusted odds ratios (AORs) with 95% confidence intervals (CIs) and two-sided *p*-values. In addition, we developed an interpretable risk score using a Framingham-style approach: model coefficients were scaled and summed to produce a point-based scoring system that estimates the probability of continued telehealth use [29]. Model performance was evaluated using survey-weighted sensitivity, specificity, and the area under the receiver operating characteristic curve (AUROC). All analyses, including the application of replicate weights, were conducted in Stata 14.2 (College Station, TX, USA).

### 2.6. Machine Learning Model Development

To compare a traditional, point-based scoring approach with contemporary machine learning (ML) methods for predicting willingness to continue using telehealth, we developed and evaluated three ML models: extreme gradient boosting (XGBoost 3.1.3), random forest (RF), and logistic regression (LR). These models were selected to represent complementary and commonly used modeling paradigms for tabular health data: logistic regression as a transparent linear baseline, random forest as a nonparametric ensemble method, and XGBoost as a state-of-the-art gradient-boosting approach with strong performance in structured datasets. Generally speaking, these models allow for comparison across interpretability, flexibility, and predictive performance while maintaining feasibility for clinical deployment. Model development proceeded in four sequential phases: (1) a baseline specification with default settings; (2) class-balance adjustment; (3) hyperparameter tuning with 5-fold cross-validation; and (4) clinical optimization to enhance practical applicability. Regarding class-balance adjustment, we employed class-weight adjustment during model training and optimized decision thresholds to balance recall and precision for the minority (unwilling) class. We prioritized performance metrics that are robust to imbalance, including recall, precision, F1 score, and AUROC, rather than accuracy alone. Synthetic resampling methods (e.g., SMOTE) were not applied because the data were survey-weighted and resampling could distort population representativeness. The hyperparameter values for each machine learning model are reported in Table A1. All models were trained and evaluated using identical training–test splits, cross-validation procedures, and performance metrics to ensure fair comparison under equivalent experimental conditions. Final decision thresholds were selected to strike a balance between recall and precision. For rigorous assessment, we reported standard classification metrics on both the training and test sets, including accuracy, recall (sensitivity), precision (positive predictive value), F1 score, and the area under the receiver operating characteristic curve (AUROC). This standardized reporting enables transparent benchmarking and meaningful comparison across modeling strategies.

### 2.7. Machine Learning Model Interpretation

The best-performing ML model was selected for interpretation. We computed Shapley Additive exPlanations (SHAP) values for all features. We visualized global importance using (1) a ranked feature-importance plot displaying the mean absolute SHAP value for each variable and (2) a SHAP summary (beeswarm) plot. The feature-importance plot highlights the top 10 contributors to the model’s predictions. The beeswarm plot provides a global view of how each feature influences the predicted probability across all observations. Each point represents an instance-level SHAP value, with its position indicating both the magnitude (effect size) and direction (positive or negative impact on the outcome). This approach clarifies both which features matter most and how they shape predictions, supporting transparent translation to clinical contexts.

### 2.8. Machine Learning Model Fairness Evaluation

To mitigate systematic biases that could undermine model validity and clinical utility, we incorporated fairness evaluation as a core component of model development. Assessments were conducted at both the group and individual levels, with a focus on gender and race/ethnicity.

Group fairness: We quantified disparities in predictions using standard metrics: (1) Disparate impact (DI): expressed as both the DI difference (absolute difference in favorable prediction rates between privileged and unprivileged groups) and the DI ratio (their ratio); and (2) Equal opportunity (EO): expressed as the EO difference (difference in recall/sensitivity between groups) and the EO ratio (their ratio). Consistent with standard practice, models were considered acceptably fair when the absolute difference was <0.10 and the ratio fell between 0.80 and 1.20 for these metrics.

Individual fairness: We evaluated counterfactual fairness, which requires that a model’s prediction for an individual remains stable when a protected attribute is hypothetically altered. At the same time, all other characteristics are held constant. To assess this, we generated counterfactual records by perturbing only the protected attribute(s), re-ran the model, and compared predictions. Any change attributable solely to the protected attribute was counted as a violation. We report the counterfactual flip rate (CFR). The CFR was calculated as the proportion of individuals whose predictions changed when the counterfactual was applied. Lower CFR indicates greater counterfactual fairness (i.e., prediction invariance to protected attributes). In general, CFR < 3–5% can be a reasonable target, considering that the model depends weakly on the attributes.

### 2.9. Scoring System Comparisons

Using a Framingham-style approach, we derived a traditional point-based scoring system from a multivariable logistic regression model and evaluated its performance (sensitivity, specificity, and AUROC). To compare this traditional score with ML approaches, we constructed an ML-informed score with the same number of predictors as the traditional system. Specifically, we selected the top features from the best-performing ML model based on global feature importance, then refit a parsimonious logistic regression using those predictors to generate point allocations using the same Framingham coefficient-to-points mapping. We compared (1) the overlap in selected predictors between the traditional and ML-informed scores and (2) the discrimination and operating characteristics of the two scores (sensitivity, specificity, and AUROC). All analyses were implemented in Stata 14.2 (College Station, TX, USA) and Python 3.8. *p*-values are interpreted in an exploratory context to identify associations informing score development rather than for formal hypothesis testing. Accordingly, results should be interpreted with attention to effect sizes and consistency rather than solely to statistical significance.

## 3. Results

**Figure 1:**
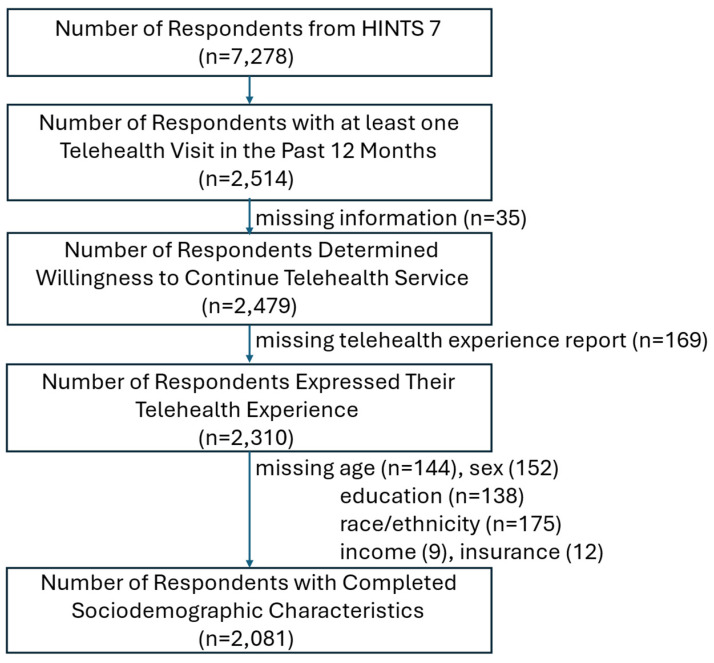
Study Flow Diagram.

HINTS 7 included 7278 respondents, of whom 2514 (33.77%) reported at least one telehealth visit in the prior 12 months. After excluding cases with missing key sociodemographic variables including age (*n* = 144), sex (*n* = 152), education (*n* = 138), race/ethnicity (*n* = 175), income (*n* = 9), and insurance status (*n* = 12) as well as telehealth experience variables (*n* = 169), the final analytic sample comprised 2081 respondents, representing an estimated 77,263,938 U.S. adults when survey weights were applied (Figure 1).

Among the respondents who had at least one telehealth visit, 1920 (91.72%) anticipated telehealth use in the future. Table 1 compares the sociodemographic and telehealth experience characteristics between individuals willing to continue and those unwilling. Sociodemographic profiles were generally similar across groups, except for age. Individuals reluctant to continue were more often younger adults (18–34 years: 42.86%) compared with those willing to continue (*p* = 0.0124).

In contrast, several telehealth experience factors were significantly associated with anticipated telehealth use. These included clinician recommendation, perceived convenience, the occurrence of technical problems, perceived quality of care, ease of access via telehealth, and respondents’ willingness to recommend telehealth to others (Table 1).

In survey-weighted multivariable logistic regression, four telehealth-experience factors were independently associated with unwillingness to continue telehealth (Table 2). Specifically, respondents who perceived telehealth as less convenient, reported technical problems, rated telehealth care quality as poor relative to in-person care, or were unwilling to recommend telehealth to others had lower odds of continuing use (Table 2). A complete model including all sociodemographic and telehealth-experience covariates is presented in Table A2.

Using a Framingham-style coefficient-to-points mapping, we developed the CONtinue TElehealth Screening Tool (CONTEST, Table 3), which demonstrated strong discrimination (survey-weighted AUROC, 0.876; 95% CI, 0.843–0.908). With a prespecified cutoff of 4.9 points (scores < 4.9 indicating higher likelihood of discontinuation), the sensitivity was 0.83, and the specificity was 0.81; the positive and negative predictive values were 0.98 and 0.70, respectively (Appendix A Table A3).

As an alternative to the point-based score, we evaluated ML methods to predict both willingness and unwillingness to use telehealth in the future. We trained three models (XGBoost, RF, and LR) and compared their performance. XGBoost demonstrated superior classification performance relative to the other approaches for anticipated telehealth use (Table 4).

**Figure 2:**
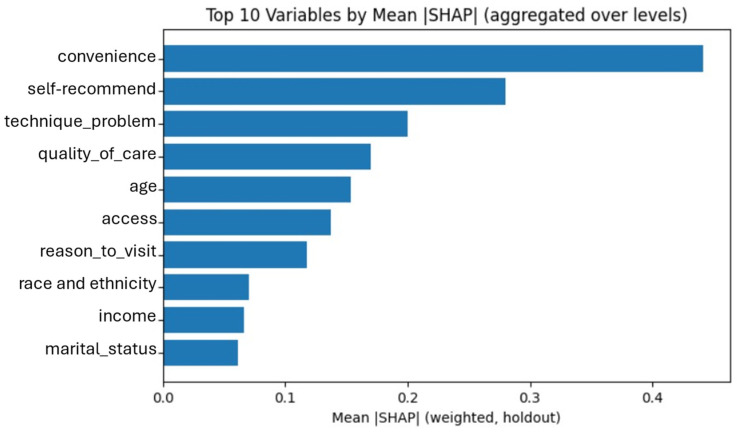
Top 10 Features Contributing to XGBoost Model Prediction.

**Figure 3:**
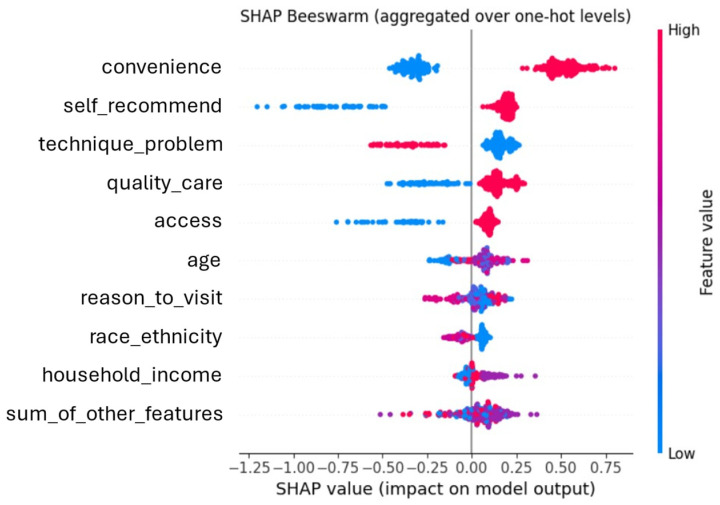
SHAP Summary Beeswarm Plot Generated by XGBoost Model.

Global interpretation and fairness assessment focused on the XGBoost model. Using SHAP values, the highest-impact features aligned with the results of the multivariable logistic regression. The factors were perceived convenience, willingness to recommend telehealth to others, technical problems, and perceived quality of telehealth compared to in-person care (Figure 2). We further visualized SHAP values using a beeswarm plot to characterize the influence of each feature across individuals (Figure 3). In this plot, higher feature values are shown in red and lower values in blue, with horizontal position indicating each feature’s contribution to the predicted outcome. For example, for convenience, lower values (blue) indicate respondents who perceived telehealth as less convenient, thereby shifting the predictions toward unwillingness to continue using telehealth. In contrast, higher values (red) shifted the predictions toward anticipated use.

In evaluating model fairness, we observed subgroup differences by sex, race, and ethnicity in the prediction of anticipated telehealth use. Overall, predictions favored male participants relative to female participants. For identifying individuals unwilling to continue using telehealth, recall was higher among Hispanic respondents, whereas precision was higher among NHB respondents (Table 5). These patterns underscore the need for caution in applying the model across subgroups and suggest potential unfairness across sex, racial, and ethnic strata. Additionally, findings from DI and EO analyses were directionally consistent (Table 6). Both DI and EO indicated measurable disparities across sex, race, and ethnicity, with inequities more pronounced for predictions of unwillingness to continue telehealth than for predictions of willingness. In individual-level counterfactual analyses, the sex counterfactual flip rate (CFR) was 0.024, indicating that reversing the sex attribute (male ↔ female) altered the model’s prediction in 2.4% of cases. The race/ethnicity CFR was 0.013, reflecting a 1.3% change in predictions. These low CFR values suggest that individual-level predictions were relatively stable under counterfactual changes to sex or race/ethnicity, consistent with counterfactual fairness at the individual level.

**Figure 4:**
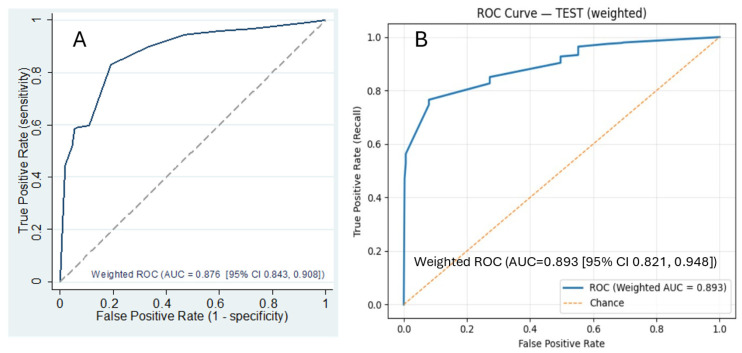
AUROC Comparisons Derived from Traditional Statistics versus ML Model. (**A**): AUROC from CONTEST score; (**B**): AUROC from four-feature XGBoost Model.

When comparing the predictive performance of the CONTEST score with the XGBoost model, the survey-weighted AUROC for CONTEST was 0.876, whereas the full-feature XGBoost model achieved an AUROC of 0.902. Because the latter used all available predictors, we conducted an additional comparison. First, starting from the complete XGBoost model, we identified the same four top features highlighted by the multivariable logistic regression. Second, we retrained XGBoost using only these four features; this model yielded a weighted AUROC of 0.893 (95% CI, 0.821–0.948), indicating performance comparable to the traditional score (Figure 4). Survey-weighted bootstrap paired tests revealed no statistically significant differences among the two models (CONTEST from logistic regression [A] vs. four-feature XGBoost AUROC [B], *p* = 0.564).

## 4. Discussions

Using nationally representative HINTS 7 data (2024), we found that approximately one-third of adults reported at least one telehealth visit in the prior year, a slight decline from the 2022 report, yet consistent with current estimates of U.S. telehealth utilization [6,30]. Among the telehealth users, nearly one in ten expressed an unwillingness to continue using telehealth in the future. Given telehealth’s role as a complementary modality within hybrid care pathways, lapses in anticipated continued use may shift demand back to in-person settings, potentially straining appointment capacity and high-acuity services.

Our multivariable analysis identified four experience-based factors that were independently associated with unwillingness to continue telehealth: lower perceived convenience, technical difficulties, perceptions of inferior care quality relative to in-person visits, and unwillingness to recommend telehealth to others. These factors cohered clinically and operationally. Convenience and technical reliability shape the friction of access, perceived quality informs trust and diagnostic confidence, and willingness to recommend is a behavioral proxy for overall acceptance [31,32]. Building on these findings, we derived the CONTEST, a simple point-based scoring system that demonstrated strong discrimination for predicting unwillingness to continue telehealth. Importantly, the cross-sectional design of this study precludes establishing temporal ordering or causal relationships between telehealth experience and anticipated future use. Telehealth experiences and anticipated use were measured at the same time point, and observed associations should therefore be interpreted as correlational rather than directional.

We further compared this traditional approach with machine learning (ML). Notably, when constrained to the same four top features, the ML model’s discrimination was similar to CONTEST, and an ML-informed four-variable point score yielded nearly identical performance. These head-to-head results highlight a practical trade-off: a parsimonious, interpretable score can perform on par with a feature-matched ML model. In contrast, a full-featured ML model may offer modest incremental gains, but at the cost of complexity. In settings where rapid bedside interpretability is paramount, CONTEST may be preferable; where infrastructure supports deployment and monitoring, ML can be advantageous, especially for ongoing performance surveillance and recalibration.

From an implementation perspective, lower precision for the minority class implies a higher false-positive rate when identifying individuals with low anticipated telehealth use. However, because the proposed tools are intended for screening or risk stratification rather than diagnosis, false positives would primarily result in additional low-risk support (e.g., technical assistance or onboarding) rather than clinical harm. This trade-off may be acceptable in settings where the cost of missed disengagement outweighs the operational burden of additional outreach.

A distinctive contribution of this study is the integrated evaluation of fairness. Group-level metrics revealed performance differences across sex, race, and ethnicity, with inequities more pronounced when predicting unwillingness to continue telehealth than when predicting willingness. At the individual level, counterfactual analyses yielded low flip rates for sex and race/ethnicity, suggesting predictions were largely stable when only a protected attribute was hypothetically altered. The apparent discrepancy between group fairness and individual counterfactual fairness highlights that fairness is a multidimensional concept. Counterfactual and group fairness capture distinct dimensions of equity and should be interpreted jointly rather than hierarchically. Additionally, models can exhibit acceptable counterfactual behavior for individuals yet still produce group-level disparities due to underlying feature distributions, structural correlates of protected attributes, or thresholding choices [33,34,35]. More importantly, clinical feedback is essential to developing fair and effective ML models, and efforts should be made to involve clinicians actively in the process [36]. Therefore, fairness assessment should combine multiple complementary metrics, and mitigation should consider involving both patients and clinicians.

Our results suggest several actionable levers to support anticipated telehealth use: (1) reduce friction to improve convenience. We may need to streamline log-in, offer device/browser compatibility guidance, and provide real-time technical support to preempt connectivity issues [31,37]; (2) assure quality: we may need to standardize virtual exam protocols, integrate peripherals where appropriate (e.g., BP cuffs, pulse oximetry), and embed decision-support to strengthen diagnostic confidence [20,38,39]; (3) build trust and advocacy: these findings suggest potential areas for clear care plans, teach-back, and follow-up options during the conversation; satisfied users who would recommend telehealth are more likely to remain engaged [21,40]; (4) target the support using CONTEST: we need to use the score to flag patients at higher risk of discontinuation and trigger tailored interventions (e.g., proactive tech navigation, longer onboarding); and (5) monitor equity: we could pair deployment with routine fairness dashboards (group and individual metrics) and evaluate whether workflow adjustments reduce observed disparities [4,41]. These operational considerations are offered as implications for future implementation and evaluation rather than interventions tested in the present study. Only under these circumstances can telehealth be robust and continue to provide satisfactory service in the long run.

This study has its strengths. We utilized a nationally representative sample, thereby enhancing the generalizability of our findings across regions and demographic groups. We present a dual-methods predictive strategy, an interpretable, point-based score, and an ML approach, with harmonized comparisons, complete model interpretation (via SHAP), and a structured fairness evaluation. Reporting training and testing performance and incorporating explainability strengthen transparency and the potential for real-world adoption.

This study also has its limitations. First, our analytic cohort included respondents with at least one telehealth encounter in the prior year; individuals without telehealth exposure were excluded, which may limit the applicability of our findings in predicting initial adoption. Second, CONTEST was derived from the full sample without a formal derivation–validation split; because it was derived and evaluated within the same analytic sample, it may have overestimated performance. Although confidence intervals were reported, external validation or resampling-based correction would strengthen generalizability and should be pursued in future studies. In addition, although survey weights were incorporated, ML models may still reflect residual confounding from unmeasured factors. Third, fairness analyses focused on sex and race/ethnicity; other sensitive or structurally influential attributes (e.g., marital status, insurance coverage, education level) were not analyzed and may reveal additional disparities. Fourth, our model interpretation emphasized feature importance and SHAP summary plots; complementary tools (e.g., partial dependence, accumulated local effects, interaction analyses) could further illuminate mechanisms. Fifth, the outcome reflects anticipated telehealth use rather than observed continuity of use. As such, CONTEST and the ML models capture attitudinal disposition rather than realized behavior, which limits their applicability for precise resource forecasting or capacity planning. Additionally, the cross-sectional design precludes causal inference regarding telehealth experience and future willingness, and dichotomizing willingness may also obscure gradations in preference. Sixth, exclusion of respondents with missing sociodemographic or telehealth experience data may have introduced selection bias. Finally, self-reported survey measures may introduce recall or social desirability bias. The outcome was based on self-reported intention rather than observed longitudinal behavior, potentially leading to an overestimation of anticipated telehealth use. Therefore, external validation in health system-level datasets is needed before clinical deployment.

Future prospective validation of CONTEST and external evaluation of the ML models across health systems and care settings are needed. Implementation studies should test score- or model-triggered interventions (e.g., technical onboarding, enhanced virtual exam protocols) and measure downstream outcomes such as completed visits, care timeliness, ED utilization, and patient-reported experience. Fairness mitigation strategies such as targeted support, threshold optimization, or fairness-aware training should be evaluated for both effectiveness and ethical acceptability.

## 5. Conclusions

Anticipated telehealth use depends on the telehealth experience, including its convenience, technical reliability, and perceived quality. Using nationally representative data, an interpretable four-factor score (CONTEST) and a feature-matched ML model achieved comparable discrimination, highlighting a practical balance between transparency and classification performance, and offering flexible deployment options. These tools can help clinicians and health systems identify individuals at risk of disengagement and target supportive interventions. Importantly, integrating predictive analytics into routine fairness monitoring is essential to ensure equitable telehealth delivery as hybrid care models continue to evolve.

## Figures and Tables

**Table 1:** Study Population Sociodemographic Characteristics and Telehealth Experience.

	Anticipated Telehealth (No) Number (wt%)	Anticipated Telehealth (Yes) Number (wt%)	*p*
	161 (8.28)	1920 (91.72)	
Age			0.0124
18–34	38 (42.86)	355 (23.01)	
35–49	35 (19.59)	514 (33.37)	
50–64	42 (20.40)	515 (27.35)	
65–74	26 (9.56)	351 (11.00)	
75+	20 (7.60)	185 (5.26)	
Sex			0.093
Male	69 (56.68)	659 (44.09)	
Female	92 (43.32)	1261 (55.91)	
Race and ethnicity			0.1078
NHW	72 (45.83)	1053 (62.31)	
NHB	29 (16.56)	294 (11.83)	
Hispanic	41 (21.95)	383 (14.96)	
Others	19 (15.66)	190 (10.90)	
Marital Status			0.0504
Single	42 (35.50)	437 (31.51)	
Married	70 (45.10)	1059 (58.48)	
Others	49 (19.40)	424 (10.01)	
Education			0.3484
Less than HS	31 (28.38)	300 (19.86)	
HS/some college	51 (38.35)	542 (39.12)	
College and above	79 (33.27)	1078 (41.02)	
Household Income			0.0578
<$50,000	75 (49.38)	661 (33.20)	
$50,000–99,999	41 (20.40)	577 (28.07)	
$100,000+	45 (30.22)	682 (38.73)	
Health Insurance			0.1053
No	21 (14.82)	136 (7.66)	
Yes	140 (85.18)	1784 (92.34)	
Recommend			0.0116
No	72 (36.71)	1045 (53.23)	
Yes	89 (63.29)	875 (46.77)	
Advice			0.0574
No	132 (72.14)	1613 (84.22)	
Yes	29 (27.86)	307 (15.78)	
Avoidance			0.7166
No	131 (79.44)	1585 (81.32)	
Yes	30 (20.56)	335 (18.68)	
Convenience			<0.0001
No	124 (88.57)	717 (39.38)	
Yes	37 (11.43)	1203 (60.62)	
Availability			0.7565
No	146 (89.88)	1732 (91.03)	
Yes	15 (10.12)	188 (8.97)	
Involvement			0.1106
No	154 (96.88)	1809 (93.51)	
Yes	7 (3.12)	111 (6.49)	
Technique			0.0013
No	97 (59.25)	1562 (80.73)	
Yes	64 (40.75)	358 (19.27)	
Quality			<0.0001
No	96 (59.76)	341 (16.60)	
Yes	65 (40.24)	1579 (83.40)	
Access			<0.0001
No	73 (43.15)	171 (7.45)	
Yes	88 (56.85)	1749 (92.55)	
Self-recommend			<0.0001
No	84 (45.75)	191 (7.18)	
Yes	77 (54.25)	1729 (92.82)	
Reason-to-visit			0.6075
Annual checkup	50 (27.52)	629 (31.79)	
Mild/Acute care	49 (30.78)	483 (25.39)	
Chronic condition	26 (15.40)	319 (17.67)	
Mental health	21 (22.33)	345 (18.02)	
Others	15 (3.97)	144 (7.14)	

**Table 2:** Independent Factors Associated with Unwillingness to Continue Telehealth using Multivariate Logistic Regression.

Factors	AOR (95% CI)	*p*
Convenience		
No	Reference	
Yes	0.08 [0.03, 0.21]	<0.001
Technique problem		
No	Reference	
Yes	2.34 [1.10, 4.97]	0.028
Quality		
No	Reference	
Yes	0.33 [0.15, 0.73]	0.008
Self-recommend		
No	Reference	
Yes	0.15 [0.05, 0.46]	0.002

**Table 3:** CONTEST Scoring System to Predict Individuals Unwilling to Use Future Telehealth Services.

Items	Score (Ranging from 0 to 10)
Telehealth is convenient	
No	0
Yes	5.1
I will recommend others to the telehealth service	
No	0
Yes	2.7
Telehealth receives the same quality as an in-person visit	
No	0
Yes	1.2
I have technical problems when using telehealth services	
No	1
Yes	0
Total Score:	

**Table 4:** Performance Metrics of Different Machine Learning Algorithms.

	XGBoost		RF		LR	
Predicting individual with anticipated telehealth use						
Weighted	Train	Test	Train	Test	Train	Test
Accuracy	0.90	0.84	0.88	0.81	0.88	0.81
Precision	0.99	0.99	0.99	0.97	0.98	0.97
Recall	0.90	0.83	0.88	0.81	0.88	0.82
F1 score	0.94	0.90	0.93	0.88	0.93	0.89
AUROC	0.96	0.90	0.97	0.88	0.83	0.79
Predicting individual without anticipated telehealth use						
Weighted	Train	Test	Train	Test	Train	Test
Accuracy	0.90	0.84	0.88	0.81	0.88	0.81
Precision	0.44	0.36	0.39	0.30	0.37	0.30
Recall	0.89	0.90	0.94	0.76	0.78	0.76
F1 score	0.59	0.51	0.55	0.43	0.50	0.43
AUROC	0.96	0.90	0.97	0.88	0.83	0.79

**Table 5:** Fairness Evaluation of Different Sub-cohort Groups.

	Female	Male	NHW	NHB	Hispanic
Predicting individual willingness to continue future telehealth services					
Accuracy	0.78	0.89	0.88	0.79	0.73
Precision	0.99	0.99	0.99	0.98	1.00
Recall	0.77	0.88	0.88	0.76	0.71
F1 score	0.87	0.93	0.93	0.86	0.83
AUROC	0.85	0.93	0.87	0.88	0.93
Predicting individual unwillingness to continue future telehealth services					
Accuracy	0.78	0.89	0.88	0.79	0.73
Precision	0.16	0.56	0.28	0.46	0.27
Recall	0.80	0.94	0.79	0.92	0.98
F1 score	0.26	0.70	0.41	0.62	0.43
AUROC	0.85	0.93	0.87	0.88	0.93

**Table 6:** Group Fairness Metrics of Different Sub-cohorts.

	Male vs. Female	NHW vs. NHB	NHW vs. Hispanic	NHB vs. Hispanic
Predicting individual willingness to continue future telehealth services				
DI Difference	0	0.01	−0.01	−0.02
DI Ratio	1	1.01	0.99	0.98
EO Difference	0.11 *	0.12 *	0.17 *	0.05
EO Ratio	1.14	1.16	1.24 *	1.07
Predicting individual unwillingness to continue future telehealth services				
DI Difference	0.40 *	−0.18 *	0.01	0.19 *
DI Ratio	3.50 *	0.61 *	1.04	1.70 *
EO Difference	0.14 *	−0.13 *	−0.19 *	−0.06
EO Ratio	1.18	0.86	0.81	0.94

*: not significant.

## Data Availability

The data presented in this study are available in HINTS 7at [URL/DOI], reference number https://hints.cancer.gov/data/download-data.aspx#H7 (accessed on 1 November 2025).

## Appendix A

**Table A1:** Final Hyperparameters for Machine Learning Models.

Model	Hyperparameters
XGBoost	n_estimators = 100; max_depth = 4; learning_rate = 0.05; subsample = 0.6
Random Forest	n_estimators = 600; max_features = ’sqrt’; max_depth = 12; min_samples_leaf = 10
Logistic Regression	Penalty = l2; c = 1.0; solver = ’saga’

**Table A2:** Factors Associated with Unwillingness to Continue Telehealth using Multivariate Logistic Regression (the full model).

Factors	AOR (95% CI)	*p*
Age		
18–34	Reference	
35–49	0.20 [0.07, 0.55]	0.002
50–64	0.14 [0.03, 0.62]	0.011
65–74	0.15 [0.04, 0.62]	0.01
75+	0.34 [0.06, 1.85]	0.205
Sex		
Male	Reference	
Female	2.08 [0.99, 4.36]	0.053
Race and ethnicity		
NHW	Reference	
NHB	2.33 [0.72, 7.50]	0.153
Hispanic	1.69 [0.58, 4.90]	0.326
Others	1.81 [0.57, 5.71]	0.308
Marital Status		
Single	Reference	
Married	1.41 [0.52, 3.85]	0.495
Others	2.64 [0.83, 8.34]	0.097
Education		
Less than HS	Reference	
HS/some college	1.20 [0.44, 3.30]	0.713
College and above	1.57 [0.55, 4.44]	0.391
Household Income		
<$50,000	Reference	
$50,000–99,999	0.66 [0.21, 2.06]	0.464
$100,000+	0.88 [0.30, 2.62]	0.821
Health Insurance		
No	Reference	
Yes	1.89 [0.43, 8.23]	0.391
Recommend		
No	Reference	
Yes	1.33 [0.67, 2.66]	0.391
Advice		
No	Reference	
Yes	1.73 [0.65, 4.61]	0.267
Avoidance		
No	Reference	
Yes	1.32 [0.54, 3.26]	0.536
Convenience		
No	Reference	
Yes	0.08 [0.03, 0.21]	<0.001
Availability		
No	Reference	
Yes	1.55 [0.53, 4.54]	0.412
Involvement		
No	Reference	
Yes	0.44 [0.11, 1.72]	0.23
Technique problem		
No	Reference	
Yes	2.34 [1.10, 4.97]	0.028
Quality		
No	Reference	
Yes	0.33 [0.15, 0.73]	0.008
Access		
No	Reference	
Yes	0.59 [0.19, 1.85]	0.354
Self-recommend		
No	Reference	
Yes	0.15 [0.05, 0.46]	0.002
Reason-to-visit		
Annual checkup	Reference	
Mild/Acute care	1.95 [0.91, 4.14]	0.083
Chronic condition	1.20 [0.40, 3.57]	0.739
Mental health	2.82 [0.78, 10.18]	0.111
Others	0.63 [0.16, 2.53]	0.506

**Table A3:** Performance Metrics of CONTEST Using Different Thresholds.

Cutoffs	Sensitivity	Specificity	PPV	NPV
0	1	0	0.92	1
1	0.99	0.06	0.92	0.60
1.2	0.97	0.27	0.94	0.57
2.2	0.97	0.30	0.94	0.56
2.7	0.96	0.40	0.95	0.53
3.7	0.94	0.53	0.96	0.54
3.9	0.90	0.67	0.97	0.63
4.9	0.83	0.81	0.98	0.70
5.1	0.61	0.89	0.98	0.83
6.1	0.60	0.89	0.98	0.83
6.3	0.59	0.94	0.99	0.83
7.3	0.58	0.94	0.99	0.83
7.8	0.58	0.94	0.99	0.83
8.8	0.56	0.95	0.99	0.84
9	0.52	0.95	0.99	0.85
10	0.45	0.98	1	0.86
